# New perspectives of purple starthistle (*Centaurea calcitrapa*) leaf extracts: phytochemical analysis, cytotoxicity and antimicrobial activity

**DOI:** 10.1186/s13568-020-01120-5

**Published:** 2020-10-12

**Authors:** Ivica Dimkić, Marija Petrović, Milan Gavrilović, Uroš Gašić, Petar Ristivojević, Slaviša Stanković, Peđa Janaćković

**Affiliations:** 1 Faculty of Biology, University of Belgrade, Studentski trg 16, 11000 Belgrade, Serbia; 2 Department of Plant Physiology, Institute for Biological Research “Siniša Stanković”, National Institute of Republic of Serbia, University of Belgrade, Bulevar despota Stefana 142, 11060 Belgrade, Serbia; 3 Faculty of Chemistry, University of Belgrade, Studentski trg 12-16, 11000 Belgrade, Serbia

**Keywords:** *Centaurea calcitrapa*, Phytochemistry, Liquid chromatography–mass spectrometry, Metabolite profile, New antimicrobials, Cytotoxicity

## Abstract

Ethnobotanical and ethnopharmacological studies of many *Centaurea* species indicated their potential in folk medicine so far. However, investigations of different *Centaurea calcitrapa* L. extracts in terms of cytotoxicity and antimicrobial activity against phytopathogens are generally scarce. The phenolic profile and broad antimicrobial activity (especially towards bacterial phytopathogens) of methanol (MeOH), 70% ethanol (EtOH), ethyl-acetate (EtOAc), 50% acetone (Me_2_CO) and dichloromethane: methanol (DCM: MeOH, 1: 1) extracts of *C. calcitrapa* leaves and their potential toxicity on MRC-5 cell line were investigated for the first time. A total of 55 phenolic compounds were identified: 30 phenolic acids and their derivatives, 25 flavonoid glycosides and aglycones. This is also the first report of the presence of centaureidin, jaceidin, kaempferide, nepetin, flavonoid glycosides, phenolic acids and their esters in *C. calcitrapa* extracts. The best results were obtained with EtOAc extract with lowest MIC values expressed in µg/mL ranging from 13 to 25, while methicillin resistant *Staphylococcus aureus* was the most susceptible strain. The most susceptible phytopathogens were *Pseudomonas syringae* pv. *syringae*, *Xanthomonas campestris* pv. *campestris* and *Agrobacterium tumefaciens*. The highest cytotoxicity was recorded for EtOAc and Me_2_CO extracts with the lowest relative and absolute IC_50_ values between 88 and 102 µg/mL, while EtOH extract was the least toxic with predicted relative IC_50_ value of 1578 µg/mL. Our results indicate that all tested extracts at concentration considered as non-toxic can be one of great importance in combat towards phytopathogenic and human pathogenic strains, as well as natural sources of antimicrobials.

## Introduction

In the last few decades, there have been an increased number of resistant pathogens globally due to uncontrolled use of antibiotics for medical and agricultural purposes. On the other hand, overuse of artificial chemical pesticides has led to the formation of multidrug-resistant phytopathogenic strains. The spread of multidrug-resistant strains brings a new generation of diseases that cannot be treated with existing agents. Pesticides, which are widely used for suppression of plant pathogens as well as to insure and increase crop yields, are considered as environmental pollutants and provokers of animal and human health implications. Because of that, there are increasingly seeking for natural components as an alternative to these agents. Plants are well known as a powerful natural source of metabolites that can be used in combating resistant strains through different mechanisms (Margni et al. 2002; Moghannem et al. 2016; Shin et al. 2018).

The genus *Centaurea* is one of the largest genera in the family *Asteraceae* which contains approximately 400–700 species (Garcia-Jacas et al. 2001). It belongs to the subtribe *Centaureinae* (Cass.) Dumort of the tribe *Cardueae* Cass. Members of the genus *Centaurea* are distributed all around the world, especially in Mediterranean area and Western Asia (Sussana and Garcia-Jacas, 2007). In Serbia, 32 species are represented (Gajić 1975). Ethnobotanical and ethnopharmacological studies showed that many *Centaurea* species have been used in folk medicine for treatment of various diseases (Khammar and Djeddi 2012). *Centaurea* species had been used for medical purposes (antidiarrheal, anti-inflammatory, antirheumatic, antipyretic, antibacterial and cytotoxic) for hundreds of years. Traditionally, leaves and shoots of *C. triumfettii* All., *C. urvillei* DC. spp. *stepposa* Wagenitz, *C. pullata* L. and *C. calcitrapa* L. are used in diet, either as raw material or in processed form, and some of the species are used for making beverages and tonics (Lentini 2000; Pieroni et al. 2002; Khammar and Djeddi 2012). Species such as *C. jacea* L., *C. calcitrapa*, *C. uniflora* Turra, are used to reduce fever; *C. behen* L. and *C. calcitrapa* for treatment of jaundice; *C. cyanus* L. and *C. ornata* Willd., for improving circulation; *C. ornata* and *C. sadleriana* Janka, in veterinary medicine; *C. cyanus* and *C. jacea* for appetite improvement (Arif et al. 2004; Csupor et al. 2010; Khammar and Djeddi 2012). Phytochemical studies of *Centaurea* species have shown the presence of numerous natural compounds that exhibit different biological activities (Khammar and Djeddi 2012; Borges et al. 2013; Xie et al. 2015). The presence of natural components such as sesquiterpene lactones, phenolic acids, flavonoids and steroids favors the genus *Centaurea* over other genera within the *Asteraceae* family in terms of extended biological activities (Dumlu and Gürkan 2006).

**Fig. 1:**
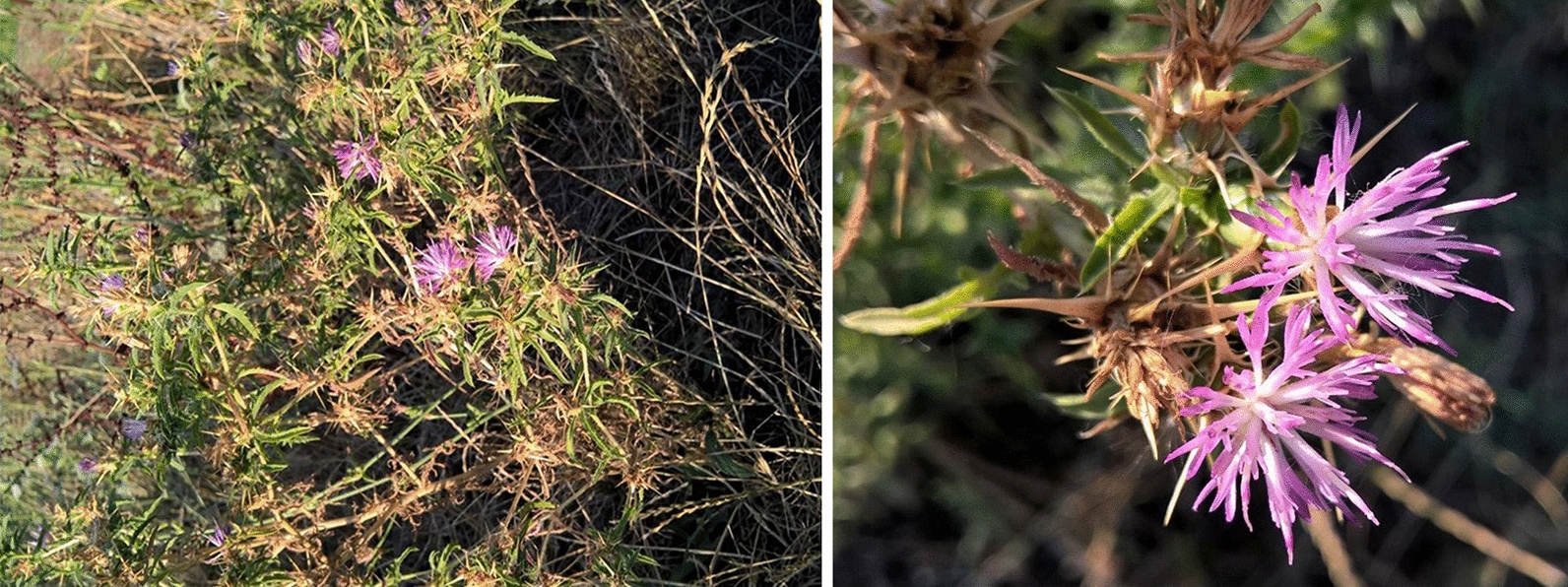
*Centaurea calcitrapa* L. (village Sakule, Opovo municipality, the South Banat District, Vojvodina province, Serbia)

*C. calcitrapa* is a biennial, herbaceous plant up to 60 cm high, commonly known as purple starthistle (Fig. 1). Flower heads (capitulum inflorescence) of this species are sessile, arranged laterally and at the top of the shoot with many bright purple-red tubular flowers. Involucre is ovoid, consisting of many phyllaries of which external are with long, yellow, terminal spine distinctive for this species. It is widespread in the western and southern parts of central Europe, North Africa and Western Asia to Northwestern India. It grows along roads, in waste places, between the rails and prefers rocky places, arable land, sunny and warm slopes (Mohlenbrock 2015). In the Southern Italy (Vulture area), young shoots of *C. calcitrapa* are consumed, as boiled and fried in mixtures with other weedy non-cultivated herbaceous plants (Pieroni et al. 2002). Traditionally, it was used for the treatment of ophthalmia, common fever, jaundice, digestive and skin disorders (Sarker et al. 2005; Csupor et al. 2010). Previous investigations on different extracts from this plant have shown certain bioactivity potential. A strong antioxidant activity has been reported for aqueous and methanol (MeOH) extracts and significant cytotoxic activity for the MeOH extract on HeLa (human cervix adenocarcinoma) and Vero (epithelial cells of African green monkey kidney) cell lines (Erol-Dayi et al. 2011). The MeOH extract has shown a strong antibacterial activity towards certain pathogens including species of *Bacillus*, *Pseudomonas*, *Staphylococcus*, *Streptococcus*, *Salmonella*, *Enterobacter*, *Enterococcus, Acinetobacter,* and *Escherichia* genera (Toribio et al. 2004; Soumia et al. 2014; Moghannem et al. 2016). Several phytochemical studies revealed sterols, sesquiterpene lactones and their closely related group of triterpenoids, bisabolenes, lignans and flavonoids as constituents of *C. calcitrapa* extracts (Karawya et al. 1975; Dawidar et al. 1989; Al-Easa and Rizk 1992; Marco et al. 1992; Formisano et al. 2012; Bruno et al. 2013; Kitouni et al. 2015).

To the best of our knowledge, there are no study reports of the cytotoxicity on MRC-5 cells and antimicrobial activity of *C. calcitrapa* extracts, particularly towards phytopathogenic bacteria*.* The main objective of the present study was to determine and evaluate phenolic profiles and antimicrobial potential of different *C. calcitrapa* leaf extracts against *Candida albicans*, as well as on human and phytopathogenic bacterial strains. In addition, cytotoxicity of different extracts was analyzed. Multidisciplinary approach was applied as an important tool for proposing new biological antimicrobial products that positively affects human health and reduces antibacterial resistance.

## Material and methods

### Plant material

Aerial parts of *C. calcitrapa* were collected during the flowering season from a village Sakule, (Opovo municipality, the South Banat District, Vojvodina province, Serbia, N 45° 08′ 14.5″, E 20° 27′ 55.8″) in July 2015. The plant material was authenticated, following professional literature for determination (Gajić 1975; Javorka and Csapody 1975). The voucher specimen was deposited in the Herbarium of the University of Belgrade—Faculty of Biology, Institute of Botany and Botanical Garden “Jevremovac” (BEOU 17509).

### Indicator strains and growth conditions

Antimicrobial activity of different extracts was tested against one yeast (*Candida albicans* ATCC 10231), five human opportunistic and pathogenic bacterial strains (*Escherichia coli* ATCC 25922, *Enterococcus faecalis* ATCC 29212, *Listeria monocytogenes* ATCC 19111, *Staphylococcus aureus* ATCC 25923 and methicillin resistant *Staphylococcus aureus* (MRSA) ATCC 33591); and nine phytopathogens (*Agrobacterium tumefaciens*, *Erwinia amylovora* NCPPB 683, *Pseudomonas syringae* pv. *aptata* (P16, P29, and P49), *Pseudomonas syringae* pv. *syringae* GSPB 1142, *Xanthomonas campestris* pv. *campestris* NCPPB 583, *Xanthomonas arboricola* pv. *juglandis* (IZB 320 and IZB 321). For differential strains growth, Luria–Bertani medium (LB) (Thermo Fisher Scientific, USA) was used for all strains, with the exception for *L. monocytogenes* and *C. albicans* which were cultured in Brain Heart Infusion broth (BHI) (HiMedia, India) and in Trypticase soy broth (TSB) (LAB M Ltd., UK), respectively. The human and phytopathogenic strains were cultured overnight at 37 °C and 30 °C, respectively. All reference strains used belonged to the Department of Microbiology, Faculty of Biology, University of Belgrade, while phytopathogenic strains, previously identified, belong to the collection of the Institute for Plant Protection and Environment, Belgrade, Serbia. All strain suspensions for antimicrobial testing were adjusted to McFarland standard turbidity (0.5), which corresponds to 10^7^–10^8^ CFU/mL.

### Cell culture

Fetal lung fibroblasts MRC-5 cells (ATCC CCL-171) were grown in RPMI 1640 medium supplemented with 10% FBS, 100 U/mL penicillin/streptomycin and 2 mM l-glutamine. Cells were maintained in a humidified atmosphere containing 5% CO_2_ at 37 °C and subcultured at 90% confluence, twice each week, using 0.05% trypsin–EDTA. Cell viability was determined by the trypan blue dye exclusion method.

### Extraction of phenolic compounds

Extraction of specialized metabolites was performed by maceration combined with ultrasonic extraction. Plant material, 10 g of air-dried *C. calcitrapa* leaves, was milled into powder for 5 min using a laboratory mill and submerged in 150 mL of a given solvent or solvent mixture: methanol (MeOH), 70% ethanol (EtOH), ethyl-acetate (EtOAc), 50% acetone (Me_2_CO) and dichloromethane: methanol (DCM: MeOH = 1: 1). Five different samples were then ultrasonicated for 30 min in the ultrasonic bath at 25 °C, and filtered through a syringe filter (PTFE 120 membrane, 0.45 µm, Supelco, Bellefonte, PA, USA), evaporated to dryness (45 °C) using rotary vacuum evaporator and extracts were dissolved in MeOH at final concentration of 30 mg/mL. The obtained methanolic solutions were stored at − 20 °C prior to analysis. The extraction yield of obtained extracts was determined according to the equation described in Janaćković et al. (2019) and expressed in percentages as a quotient of dry weight of the extracts and mass of dried plant material.

### UHPLC-LTQ Orbitrap-MS^4^

For the analysis, a 1000 mg/L stock solution of a mixture of phenolic compounds (Sigma-Aldrich Steinheim, Germany) was prepared by dissolving in MeOH (HPLC grade, Merck Darmstadt, Germany). Working solutions (0.025; 0.050; 0.100; 0.250; 0.500; 0.750 and 1.000 mg/L) were prepared by diluting the stock solution with mobile phase (acetonitrile (ACN): H_2_O = 1:1). Calibration curves were obtained by plotting the peak areas of the standards against their concentration. Ultra-pure water (Thermofisher TKA, MicroPure water purification system, 0.055 115 µS/cm) was used to prepare standard solutions and blanks. The analytical separation was performed on a Syncronis C18 column (50 × 2.1 mm, 1.7 μm particle size; Thermo Fisher Scientific, USA). The mobile phase consisted of (A) 0.1% MS grade formic acid (Merck Darmstadt, Germany) aqueous solution and (B) 0.1% formic acid in MS grade ACN (Merck Darmstadt, Germany). The gradient program was as follows: 0.0–1.0 min, 5% B; 1.0–14.0 min, 5–95% B; 14.0–14.1 min, 95–5% B; 14.1–20.1 min, 5% B. The injection volume for all samples was 5 μL and the flow rate was 275 μL per min. The UHPLC system was coupled to a linear ion trap and Orbitrap hybrid mass spectrometer (LTQ Orbitrap XL) equipped with a heated-electrospray ionization probe (HESI-II, Thermo Fisher Scientific, Germany). The system was operated in negative ionization mode with the following conditions: source voltage at 5 kV, capillary voltage at – 40 V, tube lens voltage at – 80 V, capillary temperature at 275 °C, sheath and auxiliary gas (N_2_) with a flow rate of 42 and 11 arbitrary units. The mass spectra were recorded from 100 to 900 m/z. Collision-induced dissociation (CID) was used for the study of fragmentation of the tested compounds. The normalized collision energy of the CID cell was constant and was 35 eV (Banjanac et al. 2017).

Phenolics were identified and quantified by comparison with the retention time, accurate mass and the mass spectra of standard compounds. Tentative identification of unknown compounds (in the absence of standards) were done on the basis of their monoisotopic mass (obtained by full scan analysis) and MS^4^ fragmentation and confirmed using previously reported NMR and MS fragmentation data about *Centaurea* species found in literature (Flamini et al. 2001; Forgo et al. 2012; Bakr et al. 2016; Zengin et al. 2018). ChemDraw software (version 12.0, CambridgeSoft, Cambridge, MA, USA) was used to calculate accurate mass of compounds of interest. In this way, molecular formulas of unknown compounds were obtained, while their identification was suggested according to specific MS fragmentation data.

### Principal component analysis

Principal component analysis (PCA) was performed using the PLS Toolbox software package (Eigenvector Re- search, Inc., Manson, WA, USA) for MATLAB (Version 7.12.0 R2011a), PCA was carried out as an exploratory data analysis by using singular value decomposition (SVD) algorithm.

### Antimicrobial assays

#### Well diffusion test

The initial screening test of antimicrobial activity of different extracts was determined by a modified well-diffusion method as described by Dimkić et al. (2016). Sterile molds for the wells were placed on the Plate Count (PC) Agar (bioMérieux, France) which was used as the solid medium and 6 mL of LA/TSA/BHI soft agar, previously inoculated with 60 µL (McFarland 0.5 turbidity) of the appropriate strain, was uniformly spread on the PC agar. After the soft agar solidification, into the resulting wells 20 µL of each extract (30 mg/mL) was added to the wells. As a negative control, MeOH was used, while fungicide nystatin and antibiotic vancomycin (both in 200 µg/mL concentrations) were used as positive controls. The Petri plates were incubated for 24 h at the optimal temperature (30 °C and 37 °C) for indicator strains. Diameter of growth inhibition zones was expressed in mm.

#### MIC assay

To determine the minimum inhibitory concentration (MIC) and minimum bactericidal concentration (MBC) for different *C. calcitrapa* leaf extracts, the broth microdilution method as described by Dimkić et al. (2016) was used. Two-fold serial dilutions with LB medium in 96-well microtiter plates were performed. The concentrations of tested extracts were in the range from 8–1000 µg/mL. Besides a negative control (bacterial growth control), a sterility control, and a control for the solvent (MeOH), the antibiotics streptomycin and vancomycin (Sigma Aldrich, Steinheim, Germany) were also tested as positive controls. Concentration of the antibiotics was in the range from 1–400 µg/mL, while the solvent (absolute methanol) was 10%. All dilutions were done in triplicate. Each well, except for the sterility control, was inoculated with 20 µL of bacterial culture (approx. 1 × 10^7^ CFU/mL), reaching a final volume of 200 µL. Lastly, 22 µL of resazurin [Resazurin Sodium Salt TCI, Belgium (> 90% (LC) C_12_H_6_NNaO_4_ = 251.17 g/mol)], at a final concentration of 675 µg/mL, was added to each well. The plates were incubated overnight at 30 °C and 37 °C for phytopathogenic and human pathogens, respectively. All tests were performed in a lighted environment, but the plates were incubated in the dark. The assay is based on the ability of metabolically active cells to reduce non-fluorescent resazurin to pink and fluorescent resorufin and finally to colorless dihydroresorufin by enzymes oxidoreductases within viable cells (Karuppusamy and Rajasekaran 2009). The lowest concentration which showed no change in color was defined as the MIC. MBC was determined by sub-culturing the test dilutions from each well without color change on agar plates and incubating for 24 h. The lowest concentration without bacterial growth was defined as the MBC value. The results are expressed in µg/mL.

### Cytotoxicity assay

The cytotoxic effect of the plant extracts was assessed by modified MTT assay (Mosmann 1983). Briefly, MRC-5 cells were seeded into 96-well plates at a density of 2 × 10^4^ cells/well and incubated overnight at 5% CO_2_, 37 °C. After forming a monolayer, the medium was removed and replaced with fresh medium containing test substances (concentration range was 3.9–250 µg/mL) and incubation continued for 24 h. Next day, MTT solution (final concentration 500 µg/mL) was added and cells were incubated for an additional 3 h. The formed formazan crystals were dissolved in DMSO. Cell viability was determined by measuring absorbance (A) at 570 nm using a microplate reading spectrophotometer (Multiskan FC, Thermo Scientific, Shanghai, China). Survival percentage (S) was calculated using the following formula:

$$S\left( \% \right) = 100*\left( {\frac{{A_{test\, substance} }}{{A_{control} }}} \right)$$

The concentration of the tested compound that exerts half of its maximal inhibitory effect of cell viability (IC_50_) was also determined. Quest Graph™ IC_50_ Calculator (AAT Bioquest Inc. 2009) calculator was used to generate absolute and relative IC_50_ values for each extract. The analysis of variance was supported by the Kolmogorov–Smirnov test for the normality of residuals and obtained data were subjected to the variance analysis (One-way ANOVA). The means separation of cell survival values were accomplished by Tukey’s HSD (honest significant difference) test. The significance level was evaluated at *P* < 0.05. Statistical analyses were conducted using general procedures of STATISTICA v.7 (StatSoft, Inc.) and IBM SPSS Statistics v.20 (SPSS, Inc.).

## Results

### Extraction yield

In this study, the influence of different solvents (MeOH, 70% EtOH, EtOAc, 50% Me_2_CO and DCM: MeOH = 1: 1) on the yield of extraction were examined. The yields of extraction by obtained extracts increased in the following order: EtOAc < DCM: MeOH < MeOH < 70% EtOH < 50% Me_2_CO. The lowest yield was obtained by EtOAc (9.4%). Similar yield was observed for 70% EtOH and 50% Me_2_CO extract (23.3% and 23.8%, respectively). Moderate yield was scored for MeOH and DCM: MeOH (16.6% and 13.3%, respectively).

### UHPLC-LTQ Orbitrap MS^4^

Incorporating LC–MS^4^ in an offline fashion as demonstrated in current study, allowed the identification of 55 plant metabolites: as 30 phenolic acids and their derivatives, as well as 25 flavonoid glycosides and aglycones (Table 1). Sixteen compounds were confirmed using the available commercial standards (Additional file 1: Table S1), while the others were tentatively identified by the examination of exact masses of unknown compounds and its MS^4^ fragmentations. The yields of quantified phenolics in the tested extracts can be represented as follows: EtOAc < MeOH < DCM: MeOH < EtOH < Me_2_CO. The most abundant phenolic acids in investigated extracts were hydrocinnamic acid derivatives, which were found in free form and as glycosides, and also as quinic acid esters. The phenolic acids and their derivatives (**1**–**30**) share a common fragmentation pathway based on loss of the CO_2_ group resulting in [M –H–CO_2_]^−^, –44 Da (Ristivojević et al. 2015). Phenolic acid derivatives (**11**–**13** and **15**), showed the main fragmentation mechanism based on homolytic cleavage of the ester moiety yielding a negative product ion of corresponding phenolic acid. For example, compound **18** showed a molecular ion at *m/z* 355 and was assigned to be the deprotonated molecule [M − H]^−^ of feruloyl hexoside. The MS^2^ base peak of compound **18** at 193 m*/z* (deprotonated ferulic acid) was formed by loss of hexosyl group—162 Da. MS^3^ and MS^4^ base peaks at 149 and 134 m*/z* corresponding to further loss of CO_2_ (18 Da) and methyl group (15 Da), respectively.

**Table 1:** Proposed metabolites in five different *C. calcitrapa* extracts using UPLC–MS/MS^4^ analysis

No	*t*_R_, min	Compound name	Molecular formula, [M–H]^–^	Calculated mass, [M–H]^–^	Exact mass, [M–H]^–^	Δ mDa	MS^2^ Fragments, (% Base Peak)	MS^3^ Fragments, (% Base Peak)	MS^4^ Fragments, (% Base Peak)
*Phenolic acids and derivatives*									
1	2.35	Gallic acid^*a*^	C_7_H_5_O_5_^–^	169.01425	169.01476	− 0.51	**125**(100)	96(14), 79(4), **81**(100), 9741), 107(5)	ND
2	3.98	Dihydroxybenzoyl hexoside	C_13_H_15_O_9_^–^	315.07216	315.07303	− 0.87	109(13), 152(45), **153**(100), 163(9), 165(12)	108(4), **109**(100)	65(14), **81**(100)
3	4.32	Protocatechuic acid^*a*^	C_7_H_5_O_4_^–^	153.01933	153.02006	− 0.72	**109(**100), 110(5)	**65**(100), 75(8), 81(43), 227(11)	ND
4	4.52	Dihydroxybenzoic acid pentosyl-hexoside	C_18_H_23_O_13_^–^	447.11442	447.11586	− 1.45	**152**(100), 163(65), 177(35), 179(32), 207(35)	**108**(100), 124(3)	ND
5	4.60	Caffeoyl-quinic acid isomer	C_16_H_17_O9^–^	353.08781	353.08892	− 1.12	135(6), 179(31), 180(3), **191**(100), 192(5)	109(28), 111(41), **127**(100), 171(26), 173(69)	81(7), 83(17), **85**(100), 99(33), 109(28)
6	4.64	Feruloyl-quinic acid hexoside isomer 1	C_23_H_29_O_14_^–^	529.15628	529.16127	− 4.99	173(62), **191**(100), 193(10), 337(17), 367(74)	85(81), 93(51), 99(21), 109(20), **127**(100)	81(28), **83**(100), 85(41), 99(20), 109(27)
7	4.71	Caffeoyl-hexaric acid	C_15_H_15_O_11_^–^	371.06199	371.06311	− 1.13	163(15), 191(17), **209**(100), 323(10), 325(17)	85(24), **191**(100)	**85**(100), 111(6), 147(12), 173(8)
8	4.83	Caffeoyl-quinic acid hexoside	C_22_H_27_O_14_^–^	515.14063	515.14317	− 2.54	191(26), **323**(100), 324(13), 341(12), 353(13)	133(4), **161**(100)	117(18), **133**(100)
9	5.15	Feruloyl-hexaric acid	C_16_H_17_O_11_^–^	385.07764	385.07870	− 1.07	**191**(100), 209(5), 367(5)	**85**(100), 129(7), 147(14), 173(4)	**57**(100)
10	5.17	Coumaroyl-quinic acid isomer 1	C_16_H_17_O_8_^–^	337.09289	337.09357	− 0.67	119(6), **163**(100), 164(7), 173(4)	**119**(100)	ND
11	5.24	Chlorogenic acid^*a*^	C_16_H_17_O_9_^–^	353.08781	353.08813	− 0.32	179(3), **191**(100)	**85**(100), 93(57), 111(35), 127(89), 173(63)	ND
12	5.26	Feruloyl-quinic acid hexoside isomer 2	C_23_H_29_O_14_^–^	529.15628	529.15889	− 2.62	175(29), **191**(100), 193(8), 367(70)	85(95), 93(70), 111(28), **127**(100), 173(53)	81(25), 83(26), **85**(100), 99(37), 109(39)
13	5.27	*p*-Coumaric acid^*a*^	C_9_H_7_O_3_^–^	163.04007	163.04072	− 0.65	**119**(100)	**119**(100), 91(29)	ND
14	5.29	Coumaroyl hexoside	C_15_H_17_O_8_^–^	325.09289	325.09305	− 0.16	119(9), **163**(100), 164(8), 279(5)	**119**(100)	ND
15	5.38	*p*-Hydroxybenzoic acid^*a*^	C_7_H_5_O_3_^–^	137.02442	137.02493	− 0.51	**93**(100), 94(7), 109(3)	ND	ND
16	5.47	Feruloyl-quinic acid isomer 1	C_17_H_19_O_9_^–^	367.10346	367.10347	− 0.01	134(6), **193**(100), 194(4)	**134**(100), 149(21)	**106**(100)
17	5.53	Gentisic acid^*a*^	C_7_H_5_O_4_^–^	153.01933	153.01991	− 0.57	**109**(100), 110(7)	**81**(100), 90(20), 95(12), 168(13), 198(15)	ND
18	5.54	Feruloyl hexoside	C_16_H_19_O_9_^–^	355.10346	355.10367	− 0.21	191(8), **193**(100), 194(3)	134(73), **149**(100), 178(69)	**134**(100)
19	5.76	Aesculetin^*a*^	C_9_H_5_O_4_^–^	177.01933	177.01989	− 0.56	131(5), 133(18), 134(9), **135**(100), 147(4)	**91**(100), 93(12), 107(96), 180(10), 195(10)	ND
20	5.76	Caffeic acid^*a*^	C_9_H_7_O_4_^–^	179.03498	179.03552	− 0.54	89(3), 133(2), **135**(100), 136(8)	93(15), 106(6), **107**(100), 117(24), 135(35)	ND
21	5.89	Coumaroyl-quinic acid isomer 2	C_16_H_17_O_8_^–^	337.09289	337.09279	0.10	163(5), 173(11), **191**(100), 192(3)	**85**(100), 93(50), 111(38), 127(90), 173(62)	**53**(100), 75(76), 100(85), 113(85), 176(85)
22	6.18	Feruloyl-quinic acid isomer 2	C_17_H_19_O_9_^–^	367.10346	367.10277	0.69	173(25), **191**(100), 192(4), 193(5)	**85**(100), 93(61), 111(36), 127(82), 173(65)	**57**(100), 70(19), 81(19), 167(22)
23	6.30	Coumaroyl-quinic acid isomer 3	C_16_H_17_O_8_^–^	337.09289	337.09246	0.43	163(4), **191**(100)	**85**(100), 93(55), 111(43), 127(95), 173(71)	ND
24	6.49	Feruloyl-quinic acid isomer 3	C_17_H_19_O_9_^–^	367.10346	367.10371	− 0.26	**191**(100), 192(3), 193(3)	**85**(100), 93(70), 111(33), 127(96), 173(68)	**57**(100), 80(28), 148(31), 157(27), 162(37)
25	6.82	Ferulic acid^*a*^	C_10_H_9_O_4_^–^	193.05063	193.05092	− 0.29	134(89), **149**(100), 178(74)	**134**(100)	ND
26	6.82	Feruloyl pentoside	C_15_H_17_O_8_^–^	325.09289	325.09482	− 1.93	**193**(100)	**134**(100), 149(40), 178(10)	ND
27	6.89	Feruloyl-isocitric acid	C_16_H_15_O_10_^–^	367.06707	367.06703	0.04	111(16), 155(8), **173**(100), 191(20), 321(12)	**111**(100), 129(9), 155(32)	ND
28	7.62	Caffeoyl-feruloyl-quinic acid isomer 1	C_26_H_25_O_12_^–^	529.13515	529.13590	− 0.75	173(35), 193(6), 335(20), 349(4), **367**(100)	**173**(100), 193(20)	59(10), 71(30), **93**(100), 111(44), 155(19)
29	7.72	Dihydroxybenzoyl-feruloyl acid hexoside	C_23_H_23_O_12_^–^	491.11950	491.11755	1.95	161(10), **315**(100), 323(30), 447(15), 459(22)	108(13), 151(10), **153**(100), 163(14), 165(19)	**109**(100)
30	7.97	Caffeoyl-feruloyl-quinic acid isomer 2	C_26_H_25_O_12_^–^	529.13515	529.13505	0.10	173(10), 193(5), 335(6), 353(7), **367**(100)	134(6), **173**(100), 193(61)	71(30), **93**(100), 109(18), 111(54), 155(20)
*Flavonoid glycosides and aglycones*									
31	5.57	Apigenin 6,8-di-*C*-hexoside	C_27_H_29_O_15_^–^	593.15119	593.15504	− 3.85	353(44), 383(23), **473**(100), 503(31), 575(10)	**353**(100), 354(6), 383(16)	297(48), **325**(100), 326(7)
32	6.56	Apigenin 8-*C*-hexoside	C_21_H_19_O_10_^–^	431.09837	431.09879	− 0.42	**311**(100), 312(16), 341(18), 385(5), 413(4)	**283**(100), 284(12)	163(48), 183(50), 211(30), 224(27), **239**(100)
33	6.67	Quercetin 3-*O*-glucoside^*a*^	C_21_H_19_O^12–^	463.08820	463.08780	0.40	300(14), **301**(100), 302(9)	151(83), **179**(100), 255(31), 257(14), 271(44)	**151**(100)
34	6.70	Quercetin 3-*O*-hexuronide	C_21_H_17_O_13_^–^	477.06746	477.06840	− 0.93	**301**(100), 302(9)	201(46), 229(39), 245(96), **255**(100), 283(60)	155(35), 199(19), 211(11), **227**(100)
35	6.72	Scutellarein 7-*O*-hexuronide	C_21_H_17_O_12_^–^	461.07255	461.07256	− 0.01	175(3), **285**(100), 286(4)	165(22), 185(23), 239(54), 241(34), **267**(100)	211(8), 223(13), **239**(100)
36	6.99	Apigenin 7-*O*-(6"-rhamnosyl)-hexoside	C_26_H_25_O_15_^–^	577.11989	577.12224	− 2.34	**269**(100), 270(12), 307(7)	149(35), 151(23), 201(24), **225**(100), 227(21)	169(30), **181**(100), 183(28), 196(20), 197(63)
37	7.10	Kaempferol 3-*O*-glucoside^*a*^	C_21_H_19_O_11_^–^	447.09329	447.09209	1.20	255(18), 284(96), **285**(100), 286(14), 327(16)	229(39), 241(31), 256(85), **257**(100), 267(45)	163(52), 187(14), 213(17), **229**(100), 239(24)
38	7.19	Isorhamnetin 3-*O*-glucoside^*a*^	C_22_H_21_O_12_^–^	477.10385	477.10352	0.33	271(9), 285(11), **314**(100), 315(31), 257(20)	243(21), 271(74), **285**(100), 286(42), 300(16)	**270**(100)
39	7.23	Apigenin 7-*O*-hexoside	C_21_H_19_O_10_^–^	431.09837	431.09827	0.10	268(15), **269**(100), 270(10), 311(3)	149(33), 197(27), 201(27), **225**(100), 269(32)	169(31), 181(55), 183(48), 196(32), **197**(100)
40	7.29	Apigenin 7-*O*-hexuronide	C_21_H_17_O_11_^–^	445.07764	445.07714	0.50	175(7), **269**(100), 270(7)	149(41), 151(24), 201(27), **225**(100), 227(21)	**181**(100), 183(82), 196(31), 197(49), 210(14)
41	7.45	Hispidulin 7-*O*-hexuronide	C_22_H_19_O_12_^–^	475.08820	475.08735	0.85	175(6), **299**(100), 300(15)	**284**(100), 285(2)	255(13), **256**(100), 284(20)
42	7.48	Jaceosidin 7-*O*-hexoside	C_23_H_23_O_12_^–^	491.11950	491.11788	1.62	313(42), 314(40), 327(13), 329(73), **476**(100)	**313**(100), 314(30), 315(12), 327(5), 343(17)	282(18), 285(84), 295(7), **298**(100), 299(10)
43	7.72	Kaempferide 3-*O*-hexuronide	C_22_H_19_O_12_^–^	475.08820	475.08846	− 0.26	175(13), **299**(100), 300(14)	**284**(100)	227(12), **255**(100), 256(17)
44	7.80	Kaempferol 3-*O*-acetyl-hexoside	C_23_H_21_O_12_^–^	489.10385	489.10605	− 2.20	255(37), **284**(100), 285(78), 286(12), 327(20)	227(14), **255**(100), 256(21)	211(67), **227**(100), 255(10)
45	8.10	Scutellarein	C_15_H_9_O_6_^–^	285.04046	285.04116	− 0.70	165(28), 223(33), 239(63), **267**(100), 268(19)	211(7), 223(33), 225(7), **239**(100)	167(7), 183(7), 195(57), **211**(100)
46	8.68	Luteolin^*a*^	C_15_H_9_O_6_^–^	285.04046	285.04019	0.27	151(33), 175(80), 199(77), 217(64), **241(**100)	197(91), **198**(100), 199(87), 212(16), 213(56)	169(44), **170**(100)
47	8.81	Nepetin	C_16_H_11_O_7_^–^	315.05103	315.05103	0.00	**300**(100), 301(11)	**216**(100), 227(78), 228(88), 255(48), 272(67)	173(21), 187(8), **188**(100), 201(25)
48	9.54	Apigenin^*a*^	C_15_H_9_O_5_^–^	269.04555	269.04539	0.16	149(49), 151(29), 201(30), **225**(100), 227(21)	169(22), 180(20), **181**(100), 183(36), 197(37)	ND
49	9.70	Kaempferol^*a*^	C_15_H_9_O_6_^–^	285.04046	285.04052	− 0.06	183(71), 211(98), 212(10), 213(8), **227**(100)	**183**(100), 199(56), 250(17)	ND
50	9.72	Chrysoeriol^*a*^	C_16_H_11_O_6_^–^	299.05611	299.05580	0.31	**284**(100), 285(11)	137(68), 212(58), **227**(100), 228(75), 255(36)	**183**(100), 199(20), 200(17), 213(5)
51	9.74	Jaceidin	C_18_H_15_O_8_^–^	359.07724	359.07734	− 0.10	**344**(100), 345(15)	**329**(100)	242(12), 270(14), 286(6), 301(73), **314**(100)
52	9.97	Jaceosidin	C_17_H_13_O_7_^–^	329.06668	329.06647	0.21	**314**(100), 315(11)	**299**(100), 300(3)	199(6), 227(9), 243(4), 255(11), **271**(100)
53	10.03	Kaempferide	C_16_H_11_O_6_^–^	299.05611	299.05637	− 0.25	**284**(100), 285(12)	227(7), **255**(100), 256(8)	ND
54	11.20	Eupatorin	C_18_H_15_O_7_^–^	343.08233	343.08217	0.16	**328**(100), 329(10)	**313**(100)	270(10), 282(43), 283(10), 285(43), **298**(100)
55	11.24	Centaureidin	C_18_H_15_O_8_^–^	359.07724	359.07681	0.43	301(10), **344**(100), 345(12)	**301**(100), 329(36)	258(6), 270(9), **286**(100)

^**a**^Confirmed using standards, the other compounds were identified according HRMS data and MSn; tR ─ retention time (min); Δ mDa ─ mean mass accuracy; "ND" ─ not detected

**Fig. 2:**
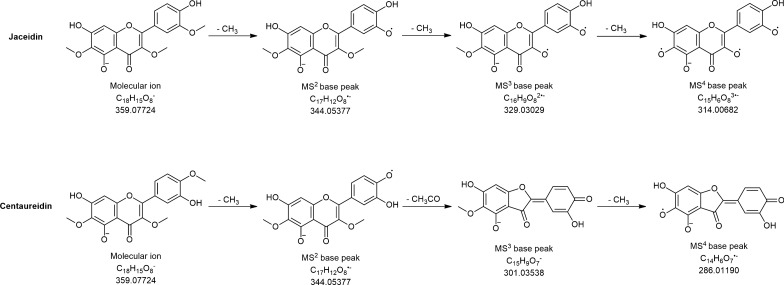
Proposed structures and fragmentation pathways of compounds **51** (jaceidin) and **55** (centaureidin)

All identified flavonoid aglycones were from the flavone subgroup (apigenin, luteolin, scutellarein, chrysoeriol, nepetin, jaceosidin, and eupatorin) and flavonol subgroup (kaempferol, kaempferide, jaceidin and centaureidin), while the glycosides were found to be flavone 7-*O* derivatives and flavonol 3-*O* derivatives. Compounds **51** and **55** with same quasimolecular ions at 359 m*/z* and retention times at 9.74 and 11.24 min, respectively, were identified by examination of its fragmentation pattern. Both compounds produced the same MS^2^ base peak at 344 m*/z* (loss of methyl group—15 Da), but different MS^3^ and MS^4^ fragmentation. In the case of compounds **55**, MS^3^ base peak at 301 m*/z* was formed by the loss of 43 Da (CH_3_ + CO), and this compound was marked as centaureidin, while the compounds **51** was marked as jaceidin, positional isomer of centaureidin. Proposed fragmentation pathways of these isomeric compounds are depicted in Fig. 2. Furthermore, eight phenolic acids (derivatives of hydroxybenzoic acids: *p*-hydroxybenzoic, protocatechuic, gallic, gentisic acid; derivatives of hydroxycinnamic acids: *p*-coumaric, ferulic, caffeic, chlorogenic acid), four flavonoid aglycones (kaempferol, apigenin, luteolin, chrysoeriol), three flavonoid glycosides (quercetin 3-*O*-glucoside, isorhamnetin 3-*O*-glucoside, kaempferol 3-*O*-glucoside) and coumarin derivative (aesculetin) were quantified (Additional file 1: Table S1). Chlorogenic acid was the most abundant of all detected compounds, with the highest amount in EtOH extract. Among the quantified hydroxybenzoic acid derivatives, *p*-hydroxybenzoic acid was the most abundant, with the highest amount in DCM: MeOH extract. The highest concentrations of protocatechuic acid, caffeic acid and *p*-coumaric acid were obtained with Me_2_CO extract, while the highest amount of ferulic acid was recorded in MeOH extract. However, with the same concentration in MeOH and EtOH extract, the highest amount of gallic acid, as well as gentisic acid, was recorded. The highest concentrations of flavonoid aglycones and glycosides were observed in Me_2_CO extract. Further, quercetin 3-*O*-glucoside, isorhamnetin 3-*O*-glucoside and kaempferol were not detected in EtOAc extract.

Due to a lack of standard compounds, the presence of compounds can be expressed in each extract through the *m/z* peak area depending of their intensities (Additional file 1: Table S2). Higher intensity of eupatorin, nepetin and jaceosidin were observed in all extracts, still their intensities were 1.12–2.36 times higher in EtOAc extract. Jaceidin, jaceosidin 7-*O*-hexoside and centaureidin were with the highest intensity in EtOAc extract, whereas intensity of jaceidin was 2.6–8.2 times higher than in all other extracts, especially comparing to the MeOH and Me_2_CO extract. The highest intensity of kaempferide and scutellarein were observed in Me_2_CO extract, while among other extracts they were least represented in EtOAc extract. Two feruloyl-quinic acid isomers were among the most represented compounds in all extracts, with the exception of EtOAc extract.

#### Principal component analysis

**Fig. 3:**
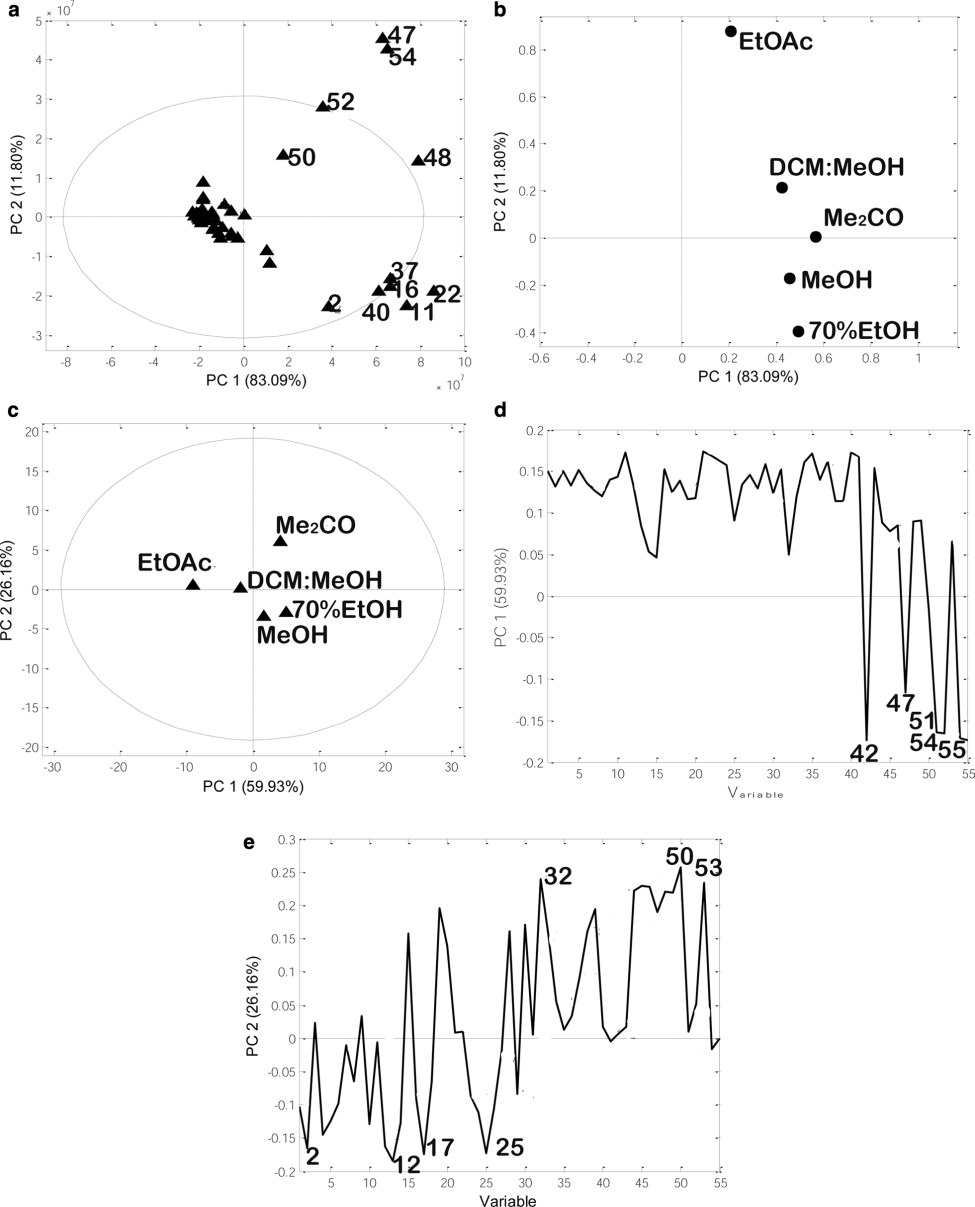
PCA analysis of tested extracts based on peak area: **a** PCs score plot, **b** loading plot, **c** PCs score plot based on extraction solvents, **d** loading plot PC1 and **e** loading plot PC2

Principal component analysis was applied to classify 55 plant metabolites from five extracts obtained using different solvents based on solvent polarity (Fig. 3). The PC1 describes 83.09%, while PC2 accounted for 11.80% of the total variability. Two-dimensional PCs showed a separation between three groups of metabolites: flavonoids such as **47**, **48**, **50**, **52**, and **54** formed one cluster, phenolic acid derivatives such as **2, 11**, **16**, **22**, **37** formed the second group of samples, while other metabolites third group. EtOAc and DCM: MeOH has the most influence on the first group of metabolites (flavonoids), while alcoholic solvents as more polar solvent showed influence on the second group of metabolites (phenolic acid derivatives). Acetone solvent showed influence on both groups of metabolites. Aforementioned results are in agreement with Additional file 1: Table S2, whereas metabolites **47**, **48**, **50**, **52** and **54** were extracted in the highest amount by ethyl acetate and acetone, while metabolites such as **2**, **11**, **16**, **22**, and **37** were extracted by alcoholic solvents and acetone. Other metabolites do not show any significant difference between five solvents. To classify metabolite profiles between five different extracts, PCA was applied: two novel PCs describing the maximum variation among the data were found. PC1 and PC2 accounted for 55.93% and 26.16%, of the total variance. Based on Fig. 3 it can be concluded that ethyl-acetate extract differs from all other extracts, while EtOH, MeOH and Me_2_CO were more similar (Fig. 3). According to the loading plot (Figs. 3d), the most important metabolites discriminating between solvents are **42**, **47**, **51**, **54**, and **55**. PC2 showed a negative loading value of metabolites **2**, **12**, **17**, and **25**, while metabolites such as **32**, **50** and **53** were positively related to PC2.

### Antimicrobial activity

#### Well diffusion

Well diffusion was used as an initial screening for the antimicrobial activity of the *C. calcitrapa* plant extracts against fourteen bacterial strains and yeast *C. albicans*. According to the obtained results (Table 2), all tested extracts, exhibited an antimicrobial effect. *C. albicans*, *X. a.* pv. *juglandis* 320 and 321 were resistant to the all extracts at the tested concentrations. Phytopathogenic isolates of *E. amylovora*, *P. s.* pv. *syringae* and *X. c.* pv. *campestris* were among the most sensitive strains to all tested extracts. Among human pathogens, the best inhibitory effect of extracts was recorded against MRSA. Also some of the isolates showed a moderate sensitivity to all extracts, while others had a high degree of sensitivity to the particular extract. The growth of MRSA and *S. aureus* was significantly inhibited by MeOH and EtOAc extracts, while the remaining extracts appeared to be moderately inhibitory. The EtOAc extract had a complete inhibitory effect on the growth of *E. coli*, while the remaining extracts caused sparse growth of this pathogen. Complete growth inhibition was observed towards strains *P. s.* pv. *aptata* P29 and P49, with the lowest activity observed for EtOH extract. A strong antimicrobial potential was recorded against *E. amylovora*, *X. c.* pv. *campestris* and *P. s.* pv. *syringae* for all of the extracts. The DCM: MeOH extract had a moderately inhibitory effect against *A. tumefaciens*, while the remaining extracts showed a high degree of growth inhibition. The best inhibitory effect against all tested strains showed EtOAc extract, with the highest zones of inhibition against *P. s.* pv. *syringae* (30.5 mm), *X. c.* pv. *campestris* (30 mm) and *A. tumefaciens* (28.5 mm). *C. albicans*, *E. faecalis*, *L. monocytogenes*, *S. aureus* and MRSA were sensitive to tested antibiotic/mycotic, while the remaining bacterial strains showed a resistance.

**Table 2:** Well diffusion assay of different *C. calcitrapa* extracts

Tested microorganisms	MeOH	EtOH	EtOAC	Me_2_CO	DCM:MeOH	Antibiotic/ Mycotic
*Candida albicans* ATCC 10231	─	─	─	─	─	13 ± 1.15
*Enterococcus faecalis* ATCC 29212	─	─	13 ± 0.29	9 ± 0.00	─	13.25 ± 0.14
*Escherichia coli* ATCC 25922	15.5 ± 0.29	12.25 ± 0.14	14 ± 0.00	12.5 ± 0.29	13 ± 0.00	─
*Listeria monocytogenes*	13.17 ± 0.17	─	18.5 ± 0.29	9.25 ± 0.14	─	17 ± 0.00
Methicillin resistant *S. aureus* ATCC 33591	21 ± 0.00	12.5 ± 0.29	20 ± 0.00	14 ± 0.58	12.5 ± 0.29	16 ± 1.15
*Staphylococcus aureus* ATCC 25923	16.17 ± 0.17	13.5 ± 0.29	21.17 ± 0.17	12 ± 0.29	13.5 ± 0.29	16.75 ± 0.43
*Agrobacterium tumefaciens*	21.83 ± 0.17	21 ± 0.29	28.5 ± 0.29	16.17 ± 0.17	14 ± 0.00	─
*Erwinia amylovora* NCPPB 683	24.5 ± 0.29	20.5 ± 0.87	26.5 ± 1.44	20.5 ± 0.29	23 ± 0.29	─
*Pseudomonas syringae* pv. *aptata* P16	10.67 ± 0.33	9.83 ± 0.17	10.75 ± 1.01	11.33 ± 0.44	12.5 ± 0.87	─
*Pseudomonas syringae* pv. *aptata* P29	16 ± 0.58	14 ± 0.58	24.75 ± 0.14	19 ± 0.00	20 ± 0.58	─
*Pseudomonas syringae* pv. *aptata* P49	13.75 ± 0.43	11.5 ± 0.29	21 ± 0.00	12.5 ± 0.00	16.5 ± 0.29	─
*Pseudomonas syringae* pv. s*yringae* GSPB 1142	19.75 ± 0.14	21 ± 0.00	30.5 ± 0.29	24.25 ± 0.43	27 ± 0.00	─
*Xanthomonas arboricola* pv. *juglandis* 320	─	─	─	─	─	─
*Xanthomonas arboricola* pv. *juglandis* 321	─	─	─	─	─	─
*Xanthomonas campestris* pv. *campestris* NCPPB 583	27 ± 0.00	22 ± 0.00	30 ± 0.00	27.25 ± 0.14	25.25 ± 0.14	─

Antibiotic—vancomycin; Mycotic—nystatin; ─ no activity. The growth inhibition zones are expressed in mm and represented as mean

values of three independent replicates ± SE

#### MIC assay

**Fig. 4:**
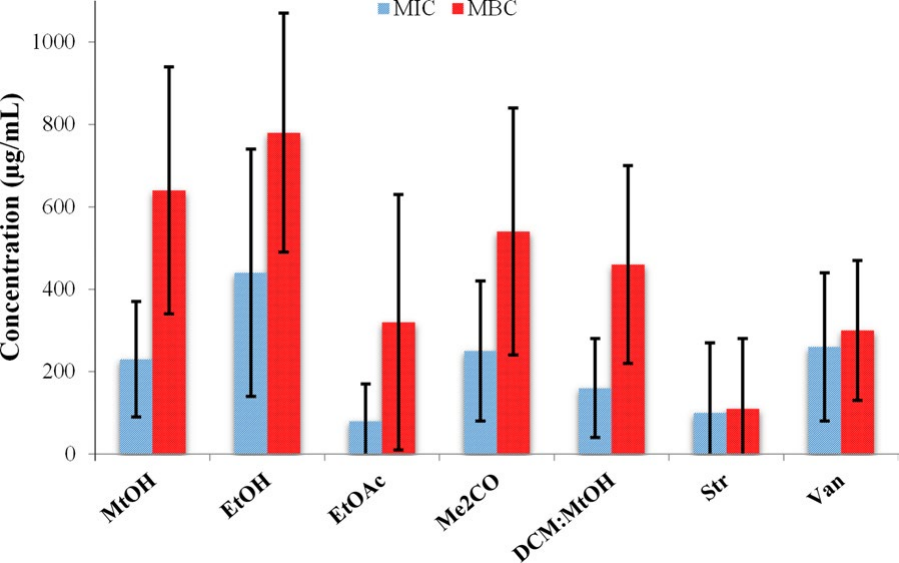
Comparison of MIC and MBC average values of the extracts towards all tested strains

After the initial screening, all extracts of *C. calcitrapa* were used in MIC assay against the most susceptible strains from the previous assay (Table 2). Among the strains used in this assay, *A. tumefaciens* and *E. coli* were more resistant. The MIC values for *A. tumefaciens* were in the range of 250–750 µg/mL depending on the extract, and for *E. coli* those values were similar, with exception for EtOH extract, without growth inhibition scored at the maximal tested concentration (Table 3). All extracts inhibited the growth of MRSA at lower concentrations than *S. aureus*, while MBC were much higher than *S. aureus*. MRSA proved to be the most sensitive isolate to all tested extracts. The best inhibitory effect against *P. s.* pv. *syringae* and *P. s.* pv. *aptata* showed EtOAc extract (MIC values of 31 µg/mL and 100 µg/mL, respectively). Differences are also evident in the bactericidal activity of the extracts against both strains, with *P. s.* pv. *syringae*, as a more susceptible strain. The MIC values of the EtOAc extract against *E. amylovora* and *X. c.* pv. *campestris* were 13 and 25 µg/mL, respectively. Also, DCM:MeOH extract showed strong activity against *X. c.* pv. *campestris* at 25 µg/mL. According to the results (Table 3), the best antimicrobial effect was recorded for EtOAc extract against all tested strains, which was in accordance with the results of the diffusion test. Based on the average MIC values towards all strain tested (Fig. 4), the antimicrobial activity of the *C. calcitrapa* extracts can be presented in following order: EtOAc > DCM: MeOH > MeOH > Me_2_CO > EtOH.

**Table 3:** Minimum inhibitory (MIC) and minimum bactericidal concentration (MBC) of *C. calcitrapa* extracts

Bacterial strains	MeOH	EtOH	EtOAc	Me_2_CO	DCM:MeOH	Str	Van
	MIC						
*E. coli* ATCC 25922	375	–	200	375	375	4.6	500
MRSA ATCC 33591	15	60	15	7.8	31	–	–
*S. aureus* ATCC 25923	94	125	16	31	62.5	9	1.6
*A. tumefaciens*	500	750	250	375	250	–	–
*E. amylovora* NCPPB 683	200	375	13	375	150	3	400
*P. syringae* pv. *aptata* P29	250	500	100	500	250	1.5	–
*P. syringae* pv. *syringae* GSPB 1142	200	200	13	150	100	3	–
*X. campestris* pv. *campestris* NCPPB 583	200	500	25	150	25	12.5	12.5
	MBC						
*E. coli* ATCC 25922	500	–	250	500	500	6	100
MRSA ATCC 33591	1000	–	1000	500	500	–	–
*S. aureus* ATCC 25923	125	250	125	125	250	25	3
*A. tumefaciens*	1000	1000	500	1000	1000	–	–
*E. amylovora* NCPPB 683	500	500	25	500	200	6	–
*P. syringae* pv. *aptata* P29	–	–	500	–	500	6	–
*P. syringae* pv. *syringae* GSPB 1142	500	500	25	200	200	6	–
*X. campestris* pv. *campestris* NCPPB 583	500	–	150	500	500	25	25

Str—streptomycin; Van—vancomycin. (–) ─ not detected in the range of tested concentrations. Values are expressed in µg/mL

### Cytotoxicity

**Fig. 5:**
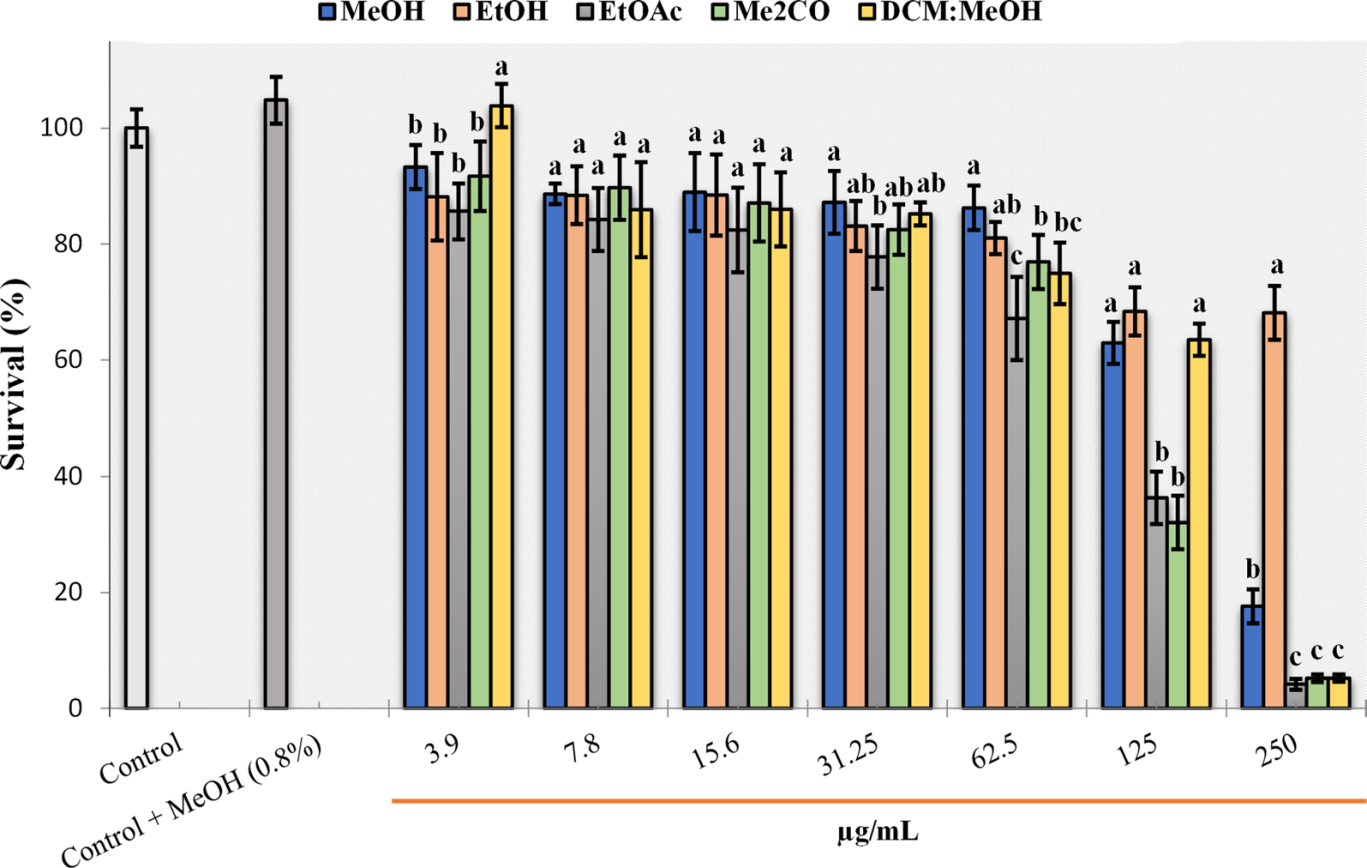
Survival of MRC-5 cells in the treatment with *C. calcitrapa* extracts. Values followed by the same letter on the histogram columns with different concentrations were not significantly different (P < 0.05) according to Tukey’s HSD test

**Fig. 6:**
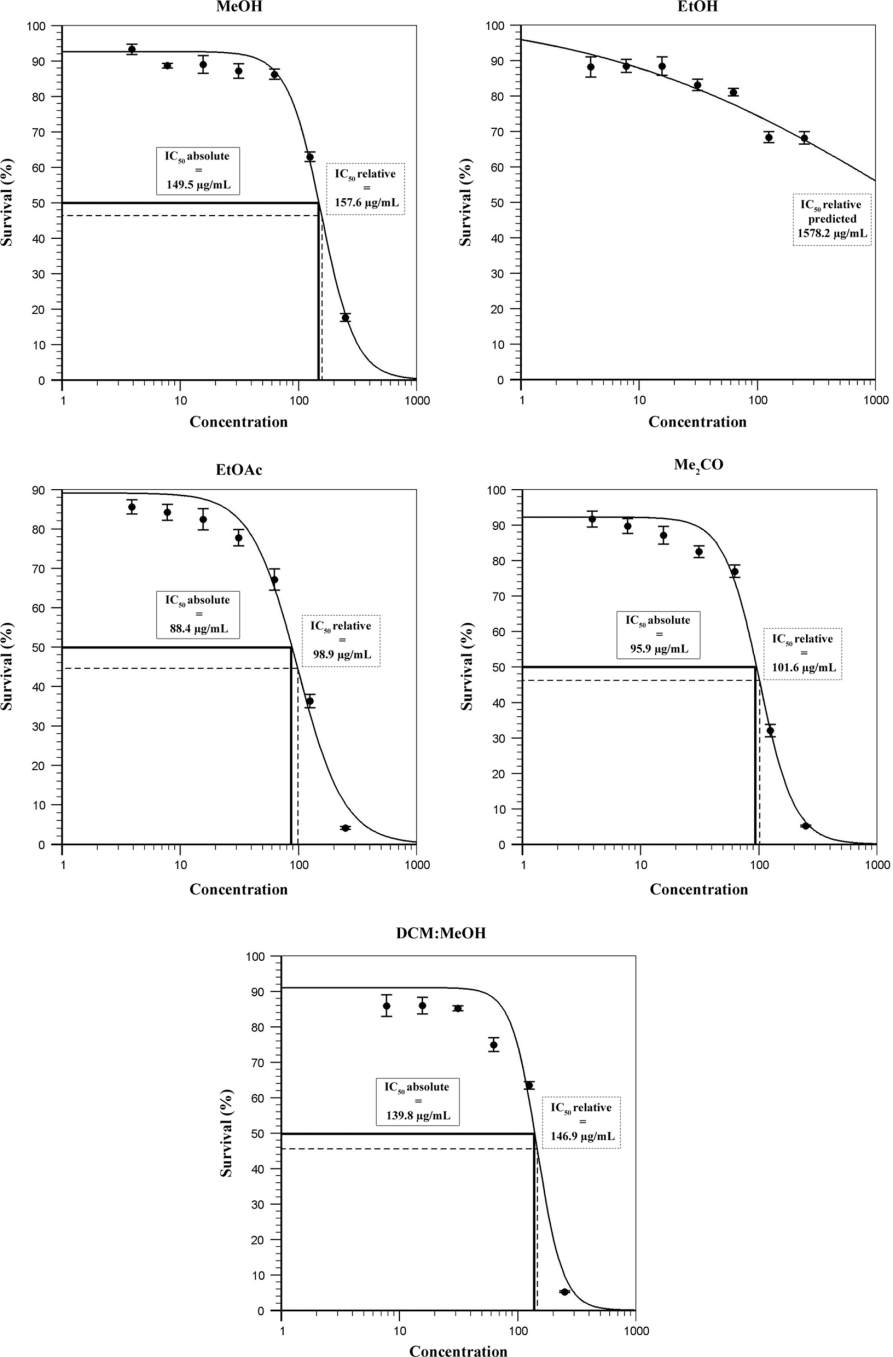
The IC_50_ absolute and relative values of the *C. calcitrapa* extracts

The results of cytotoxic activity of tested extracts were shown in Fig. 5. The viability of MRC-5 cells exposed to *C. calcitrapa* extracts showed lowest statistically insignificant cytotoxicity up to concentration of 62.5 µg/mL. The EtOAc extract at 31.25 and 62.5 µg/mL, showed slightly statistically significant elevated cytotoxicity than other extracts, with the reduction between 27 and 35% of the cell viability, respectively. On the other hand, MeOH, EtOH and DCM:MeOH extracts at concentration of 125 µg/mL showed statistically insignificant and equal cytotoxicity, while only EtOH extract at highest applied concentration (250 µg/mL) was exhibited a lowest cytotoxic effect. This finding also has been confirmed by calculated and predicted relative IC_50_ value of 1578 µg/mL. The IC_50_ absolute and relative values of the other extracts were also calculated (Fig. 6). The highest cytotoxicity was recorded for EtOAc and Me_2_CO extracts with the lowest relative and absolute IC_50_ values between 88 and 102 µg/mL. Moderate cytotoxicity was established for the MeOH and DCM: MeOH extracts with relative IC_50_ values at 158 and 147 µg/mL, respectively.

Based on a comparative analysis of the MIC and MTT test results, at concentrations considered non-toxic, the growth of methicillin-resistant *S. aureus* was successfully inhibited by all tested extracts. Also, non-toxic concentrations of EtOAc and Me_2_CO extract successfully affected the growth of MRSA and *S. aureus* at very low concentrations (Table 3). Low non-toxic concentrations of EtOAc and DCM: MeOH extracts were successful against *X. c.* pv. *campestris*, while for *E. amylovora* and *P. s.* pv. *syringae* low non-toxic MICs were obtained only for EtOAc extract.

## Discussion

Resulting extracts within this study, obtained using mixtures of water and solvent had a higher yield than the extracts obtained using pure solvents itself. It can be seen that extraction yield increases with increasing polarity of the solvent used in extraction. As EtOH and Me_2_CO were made as water-solvent mixtures, others compounds, such as sugars and organic acids, could be co-extracted and influenced the highest yield of these solvents. Karamenderes et al. (2007) examined the *n*-hexane, Chl (chloroform) and MeOH extracts of aerial parts of several *Centaurea* species and reported that the highest yield was obtained using MeOH as a solvent, which was the case in our work as well. Also, it can be spotted that Me_2_CO solvent mostly influenced the extraction of hydroxylated flavonoids, while EtOAc was the most efficient solvent for extraction of methoxylated flavonoids. The differences in the efficiency of the used solvents were also visible in the extraction of phenolic acids. More polar hydroxybenzoic acids were most extracted with EtOH and MeOH solvents. Still, all solvents used similarly influenced the extraction of protocatechuic acid, with exception of EtOAc which was the least efficient solvent. Me_2_CO and DCM: MeOH were the most efficient for extraction of *p*-hydroxybenzoic acid with similar extraction capacity. Me_2_CO solvent had the greatest influence on the extraction of hydroxycinnamic acids, *p*-coumaric and caffeic acid. Compared to the other solvents used, MeOH was proved to be the best solvent for the extraction of ferulic acid. However, water–ethanol mixture gave the most satisfactory results when it comes to extraction of chlorogenic acid. Methodology applied through using UHPLC-LTQ Orbitrap MS reveals 55 plant metabolites and fragmentation data confirmed the presence of many compounds which are revealed for the first time in *C. calcitrapa* extracts such as jaceidin and its positional isomer centaureidin. MS fragmentation data about fragmentation of jaceidin and its isomer were consistent with previously published MS data (Taamalli et al. 2015). Among the quantified flavonoids, apigenin was the most abundant in our study, which has also been identified in other *Calcitrapa* species such as *C. pallescens* Delile, *C. urvillei*, *C. affinis* Friv., *C. cyanus*, *C. chilensis* Bertero ex Hook. & Arn., *C. nigra* L., *C. virgata* Lam. (Khammar and Djeddi 2012). Many phenolics identified in *C. calcitrapa* extracts, have been isolated from numerous *Centaurea* species (Erel et al. 2011; Khammar and Djeddi 2012; Azzouzi et al. 2016). However, the chemical composition was dissimilar to this study to a certain extent. Therefore, *C. calcitrapa* can be considered as a species with high phenolic diversity. Flavonoids such as apigenin, luteolin, kaempferol, kaempferol 3-*O*-glucoside, eupatorin and jaceosidin have been previously detected in the *C. calcitrapa* (Al-Easa and Rizk 1992; Formisano et al. 2012; Kitouni et al. 2015). In the study of Öksüz et al. (1984), apigenin and jaceosidin have been isolated from all of the investigated *Centaurea* species (*C. virgata*, *C. kilea* Boiss., *C. inermiss* Velen.), whereas apigenin was the major isolated compound. Also, eupatorin, nepetin and kaempferol 3-O-glucoside found to be constituents of *C. virgata* and *C. inermis*. However, eupatorin and jaceosidin besides the above mentioned species have been found together in aerial parts of *C. grisebatchii* (Nyman) Heldr (Djeddi et al. 2008a). Nepetin and protocatechuic acid from *C. calcitrapa* leaves have been previously detected in the related species *C. melitensis* L. (Ayad et al. 2012; Khammar and Djeddi 2012). Chlorogenic acid together with protocatechuic acid, kaempferol 3-*O*-glucoside and quercetin 3-*O*-glucoside has been obtained from *C. isaurica* Hub.-Mor extracts (Flamini et al. 2004). Combination of apigenin, luteolin, kaempferol, chlorogenic acid and caffeic acid as natural compounds has also been found in aerial parts of *C. cyanus* (Litvinenko and Bubenchikova 1988). Also, nepetin, luteolin, centaureidin, jaceidin, caffeic acid and protocatechuic acid have been isolated together from *C. bracteata* (Flamini et al. 2001).

A strong inhibitory effect of EtOAc extract of numerous *Centaurea* species, except for *C. calcitrapa*, has been reported, so far, by using disk diffusion method, against many bacteria, including *S. aureus*, *E. coli* and *L. monocytogenes* (Güven et al. 2005). The inhibitory activity of MeOH extract of *C. calcitrapa* leaves against *S. aureus* (18 mm) and *E. coli* (20 mm) has been reported in Moghannem et al. (2016), which confirmed by the results obtained in our study. Also, activity of 70% MeOH extract of *C. calcitrapa* aerial parts (100 mg/mL) has been reported by disk diffusion method towards *S. aureus* and *E. coli*, whereas extract found to be inactive against *E. coli* at the tested concentration and moderately active against *S. aureus* with 13 mm in diameter (Soumia et al. 2014). Furthermore, Güven et al. (2005) investigated antimicrobial potential of EtOAc, Chl, Me_2_CO and EtOH extracts of several *Centaurea* species, except for *C. calcitrapa*, where EtOAc extract showed the best activity against tested microorganisms and was further examined with the microdilution method. Obtained MIC values against *S. aureus* at a concentration of 125 µg/mL for *C. ptosomipappoides* Wagenitz, *C. odyssei* Wagenitz, *C. ptosomipappa* Hayek, *C. kurdica* Reichardt, and 62.5 µg/mL for *C. amonicola* Hub. Mon., was higher than in our study. Antimicrobial activity of MeOH extract of capitula (diffusion method) of *C. calcitrapa* has been reported in the literature, with an inhibitory effect against certain bacterial strains including the same strains of *S. aureus* and *E. coli* as in this study (Toribio et al. 2004). While extract inhibited growth of *S. aureus* at concentration of 400 µg/mL, it was reported as inactive towards *E. coli* at the maximum concentration tested. Comparing our results with the literature data, it was evident that MeOH extract of *C. calcitrapa* leaves exhibits significantly higher antimicrobial activity, at lower concentrations, than the MeOH extracts of the capitula and 70% MeOH extract of the entire aerial parts of this species.

Antimicrobial activity of phenolics has been quite investigated on different bacterial strains. There are few reports about the structure–activity relationship of phenolics against MRSA and *E.coli*. Xu and Lee (2001) have been investigated flavonoids with different substitutions for their activity towards antibiotic-resistant bacteria and found that flavonols or flavones with more hydroxyl groups on the A and B rings, are better inhibitors of MRSA, whereas methoxyl substitutions reduce activity. Furthermore, they reported that a –OH at C3 is required for anti-MRSA activity of flavonols. Also, Alcaraz et al. (2000) highlighted that 5-hydroxylation of flavones is the one of greatest importance for their activity. All flavones found in *C. calcitrapa* extracts had a –OH at C5 position and were tri- or tetrahydroxyflavones. Furthermore, apigenin, scutellarein, luteolin and kaempferol, which are polyhydroxylated and without methoxy groups, may be responsible for the best activity of Me_2_CO extract towards MRSA, due to their highest intensities in this extract. In favor of this statement is the fact, from our study, that intensity of scutellarein was observed far more in Me_2_CO extract than in EtOAc extract, more precisely 169 times higher. Apigenin and luteolin have been reported for strong activity against the same MRSA strain used in this study, and while luteolin was active at 62.5 µg/mL, apigenin achieved remarkable activity at lower concentration of 3.12 µg/mL (Cushnie et al. 2003; Joung et al. 2016). However, in the study of Su et al. (2014) apigenin along with scutellarein and chlorogenic acid, appeared inactive towards numerous clinical and reference MRSA isolates, as well as against methicillin-sensitive *S. aureus* with MICs higher than 2000 µg/mL. Notable anti-MRSA activity has been reported for jaceosidin which bear a -OCH_3_ group at 5- and 3′ positions (Barnes et al. 2013), indicating that other methoxylated flavones from *C. calcitrapa* extracts may be potential inhibitors of MRSA and might contributed to the EtOAc extract activity, whereas they observed with highest intensities. Furthermore, nepetin a flavonoid with –OCH_3_ group at C-6 has been reported for inhibition of clinical isolate of MRSA (Talib et al. 2012). Nepetin was also found with highest intensity in EtOAc extract within our study. To the best of our knowledge there are no reports for antimicrobial activity of jaceidin and centaureidin against methicillin-resistant *S. aureus*. Skupińska et al. (2017) reported that the most active inhibitors of *E. coli* and *S. aureus* had a –OH groups at 5- and 7- positions and methoxy group at C-4′, such as kaempferide with MIC_50_ values of 22.7 µg/mL and 8.5 µg/mL, respectively. Also, authors reported that kaempferol was inactive against *E. coli*, while in the study of Wu et al. (2013) it was reactive with MIC_50_ value of 25 µg/mL, whereas authors indicated that hydroxyl group at C-4′ are necessary for the activity. In this study, kaempferide and kaempferol were more abundant in Me_2_CO extract, while eupatorin and centaureidin, which also bear a -OCH_3_ group at C-4′ were prevalent in EtOAc extract and might be responsible for its stronger activity on *E. coli* and *S. aureus* than Me_2_CO extract. In the study of Akroum et al. (2009) notable antimicrobial activity of kaempferol 3-*O*-glucoside and quercetin 3-*O*-glucoside has been recorded against *S. aureus* and *E. coli* with MIC values from 65–95 µg/mL. In line with that, flavonoid glycosides may be one of the compounds that enhance activity of extracts, especially of Me_2_CO extract, whereas they observed in the highest concentrations. Barnes et al. (2013) reported that activity of jaceosidin towards *E. coli* was higher than 128 µg/mL. Also, Allison et al. (2017) reported that antimicrobial activity of jaceosidin and jaceidin against a wild-type strain of *E. coli* was higher than 100 µM (which corresponds to values of 33.03 µg/mL and 36.03 µg/mL, respectively). Sánchez‐Maldonado et al. (2011) have been investigated structure–activity relationship of phenolic acids and found that lipophilicity has a greater impact on antimicrobial activity of hydroxybenzoic acids, than of hydroxycinnamic acids, whose lipophilicity is already established with properties of their side chains. Increasing number of hydroxyl groups decreased activity of hydroxybenzoic acid and when a –OH groups were substituted with methoxyl groups lipophilicity increased, which also enhanced the activity. *P*-hydroxybenzoic acid with only one –OH group achieved better inhibitor activity towards *E. coli* (MIC was 0.12 µg/mL) than more hydroxylated hydroxybenzoic acids. Generally, hydroxycinnamic acid achieved better activity towards *E. coli* with lower MIC values. Due to various methods used and different susceptibility of bacterial strains, results of antimicrobial activity of phenolics in the literature are often dissimilar. However, antimicrobial activity of certain phenolics, which are identified as constituents of *C. calcitrapa* extracts, has been reported towards the same strains used in this study. Gallic, caffeic, *p*-coumaric and chlorogenic acid exhibited significant inhibitor activity against *S. aureus* and MRSA (Rivero-Cruz, 2008; Özçelik et al. 2011; Dimkić et al, 2016). It was shown that flavonoids exhibit better activity than phenolic acids against *E. coli* (Rivero-Cruz, 2008; Liu et al. 2013; Wu et al. 2013; Skupińska et al. 2017). In our study, this link is also visible through the presence of flavonoids and phenolic acids in the extracts, whereas methoxylated flavonoids found with highest intensities in EtOAc extract, which expressed the best activity towards *E. coli*. In the study of Ravn et al. (1989) aesculetin was the most active among tested compounds against *A. tumefaciens* and *P. syringae* with MIC value lower than 100 µg/mL, while ferulic, caffeic and chlorogenic acid exhibited significantly less activity.

A strong antimicrobial effect of cnicin, mellitensin, salonitenolide, 11*β*, 13-dihydroxy-salonitenolide, pinoresinol, *β*-amyrin and *β*-sitosterol obtained from different sources against *S. aureus*, MRSA and *E. coli*, has been reported (Bruno et al. 2003; Bachelier et al. 2006; Djeddi et al. 2008b; Rivero-Cruz et al. 2009; Bach et al. 2011; Sen et al. 2012; Zhou et al. 2017). These specialized metabolites have also been detected in *C. calcitrapa* (Karawya et al. 1975; Al-Easa and Rizk 1992; Marco et al. 1992; Bruno et al. 2013). Also, two chalcones have been reported as constituents of *C. calcitrapa* and although they have not been investigated for their activity, it is worth mentioning that chalcones, generally, possess considerable antimicrobial activity, especially anti-MRSA activity (Dawidar et al. 1989; Alcaraz et al. 2000; Xie et al. 2015). It should be borne in mind here that the role of sesquiterpene lactones in increasing the antioxidant and antimicrobial effect of phenolic compounds is known but not fully clarified (Chadwick et al. 2013). Moghannem et al. (2016) isolated, but not completely determined, a compound from the group of sesquiterpene lactones from MeOH extract of *C. calcitrapa* leaves and examined its effect on bacterial isolates by using the microdilution method. The MIC (15.6 µg/mL) and MBC (31.25 µg/mL) values against *S. aureus* and *E. coli* were lower in regards to our results obtained with MeOH extract against the mentioned pathogens. To the best of our knowledge our study presents the first report of antimicrobial activity of *C. calcitrapa* extracts towards phytopathogenic bacteria. Phytopathogenic bacterial strains showed high sensitivity toward tested extracts. It has been reported that phenolic acids with a longer alkyl chain have a higher affinity for lipid bilayers, which may explain high susceptibility of Gram-negative bacteria (Borges et al. 2013). Moreover, hydrophobic character of methoxylated flavonoids may be responsible for the best activity of EtOAc extract against phytopathogenic bacteria, since they were observed with highest intensities in this extract.

In comparison of the total yield of quantified phenolics and prevalence of phenolics through their intensities per extract with accomplished MIC results, it can be seen that EtOAc extract, with the highest representation of methoxylated flavonoids, had the best antimicrobial effect. We can assume that other specialized metabolites such as sesquiterpene lactones, which are known to have strong antimicrobial activity, are also found in a high concentration of EtOAc extract and contributed to overall extract activity. This assumption is based on the work of Van Beek et al. (1990) who identified EtOAc extract as the best one for isolating sesquiterpene lactones. Also, the best anti-MRSA activity of Me_2_CO extract might be due to a greater portion of the polyhydroxylated flavonoids, since they are in the highest concentrations in this extract. Since it is known that chemical structures of flavonoids and phenolic acids, different substitutions, and different mechanisms of action due to their structural variability are responsible for antibacterial activity (Shin et al. 2018), it could be assumed that synergistic effect of the present compounds is responsible for the strong activity of the extracts. This assumption is based on the studies whereas quercetin and luteolin in combination expressed better activity than they were tested alone against different MRSA and *S. aureus* strains (Su et al. 2014; Usman et al. 2016).

As far as we know, this is the first report for cytotoxicity of *C. calcitrapa* extracts on the MRC-5 cell line. Erol-Dayi et al. (2011) have reported cytotoxicity for MeOH extract of *C. calcitrapa* leaves on Vero and Hela cells, whereas MeOH extract was less toxic on MRC-5 cells. Similar results showed that cytotoxicity of MeOH extract of *C. ainetensis* against human normal intestinal cells was negligible with the survival rate over 90% (El-Najjar et al. 2008). EtOAc extract of several *Centaurea* species exhibited similar toxicity on MRC-5 cells (Faschingbauer, 2019), which is in accordance with the results in this study. However, cytotoxic effects of extracts from different *Centaurea* species have been more investigated on cancer cells, than on healthy cell lines. Some flavonoids have been studied for their cytotoxicity on both cell lines. Csapi et al. (2010) indicated that Chl (chloroform) extract of *C. arenaria* (M. B. ex Willd.) showed the highest cytotoxic activity against several cancer cell lines. Moreover, they considered that flavonoids, sesquiterpene lactones and lignans are responsible for cytotoxicity. Beutler et al. (1998) examined cytotoxicity of flavones with different substitutions and reported that flavones with the best cytotoxic activity were one with a -OH groups at 5- and 3′ positions, as well as methoxyl groups at C-3 and C-4′. According to some authors, interrelation of substituents on A-ring is also important for the flavonoids cytotoxicity (Lopez-Lazaro et al. 2002). As previously mentioned, all identified flavones in this study were 5- hydroxylated, while some of them fully satisfy these structure–activity correlations such as centaureidin and eupatorin. Centaureidin has been reported as extremely cytotoxic compound towards breast, cervical and skin cancer cell lines with submicromolar IC_50_ values, while eupatorin and apigenin had expressed strong cytotoxicity against the same cell lines, but at higher concentrations (Forgo et al. 2012). Also, eupatorin and scutellarein achieved highly antiproliferative activity on MDA-MB-468 (breast carcinoma), but notably less on MCF-10A normal breast cell line with IC_50_ values of 50 µM and 42 µM, respectively (Androutsopoulos et al. 2008, 2009). Kaempferide has been reported as considerably cytotoxic on several cancer cell lines and non-toxic on human fibroblast (Nath et al. 2015). Cytotoxicity of apigenin, luteolin and chlorogenic acid towards MRC-5 cells has been reported, with IC_50_ values higher than 100 µM, 80 µM and 1.97 ± 0.11 mM, respectively (Csupor-Löffler et al. 2011; Burgos-Morón et al. 2012; Masraksa et al. 2020). Nevertheless, jaceosidin was more toxic on normal endometrial cells than on cancer cells (Lee et al. 2013). IC_50_ values of eupatorin and nepetin against Vero cells were higher than 500 µg/mL and 103.54 ± 2.82 µg/mL, respectively (Talib et al. 2012; Beer et al. 2016). Thus, different cell lines are more or less sensitive to the investigated phenols, and while they are toxic on cancer cell lines at low concentrations, on normal cell lines they exhibit this activity at high concentrations, furthermore they are considered non-toxic. Considering the rich phenolic profile of tested extracts, possible explanation for showed cytotoxicity at the highest tested concentrations may lay in the fact that phenolic compounds can act as pro-oxidants. When acting as pro-oxidant agents, phenolic compounds are capable of increasing the cellular level of reactive oxygen species (ROS) to cytotoxic levels, consequently leading to the cell death (Martín-Cordero et al. 2012). Also, higher toxicity of *C. calcitrapa* extracts on MRC-5 cells might be due to synergistic activity of present compounds in the extracts. Moreover, the highest intensities of centaureidin, eupatorin and jaceosidin in EtOAc extract probably contributed to the overall extract toxicity. In conclusion, UHPLC-Orbitrap MS revealed the rich phenolic profile of *C. calcitrapa* with highest intensities of apigenin, eupatorin, nepetin, jaceosidin, chrysoeriol and chlorogenic acid. To the best of our knowledge, this is the first report of centaureidin, jaceidin, kaempferide, nepetin, flavonoid glycosides, phenolic acids and their esters as constituents of *C. calcitrapa*. The EtOAc extract had the best antimicrobial activity. Certainly, correlation between phenolic compound content and the antimicrobial potential of the extracts was confirmed. The most sensitive human pathogen was methicillin-resistant *S. aureus*, which is probably due to a great deal of polyhydroxylated flavones with anti-MRSA activity. Higher sensitivity of phytopathogenic bacteria onto EtOAc extract, compared to others, is probably influenced by hydrophobic properties of methoxylated flavonoids, among others. All tested extracts, which showed the lowest toxicity but at the same time strong antimicrobial activity, can serve as a potential source of natural new compounds against human pathogenic strains, but also against significant phytopathogenic bacterial isolates. To the best of our knowledge, this is the first report of an evaluation of *C. calcitrapa* extracts against phytopathogenic and certain human pathogenic strains, and determination of their potential toxicity on normal human fetal lung fibroblasts. The future studies might include tests of synergism and cytotoxicity of major standard compounds individually and in combinations, as well as to provide us information of the endophytic microbial population from *C. calcitrapa,* which could be used in biological control together with the plant extracts in combat against most specific phytopathogens.

## Supplementary information

**Additional file 1: Table S1. **Quantified phenolics and their yield (mg/kg) in *C. calcitrapa* extracts. **Table S2. **Proposed metabolites and m/z peak areas in five different *C. calcitrapa *extracts using UPLC–MS/MS4 analysis.
