# Supplementary material for: New perspectives of purple starthistle (Centaurea calcitrapa) leaf extracts: phytochemical analysis, cytotoxicity and antimicrobial activity
Source: AMB Express. 2020 Oct 12;10:183. doi: 10.1186/s13568-020-01120-5 (PMC7550514; doi:10.1186/s13568-020-01120-5)
Supplement: Supplementary file 1 — Additional file 1: Table S1. Quantified phenolics and their yield (mg/kg) in C. calcitrapa extracts. Table S2. Proposed metabolites and m/z peak areas in five different C. calcitrapa extracts using UPLC–MS/MS4 analysis. [file 13568_2020_1120_MOESM1_ESM.pdf]

Additional file 1

New perspectives of purple starthistle (*Centaurea calcitrapa*) leaf extracts: phytochemical analysis, cytotoxicity and antimicrobial activity

Ivica Dimkić[1#*], Marija Petrović[1#], Milan Gavrilović[1], Uroš Gašić[2], Petar Ristivojević[3], Slaviša Stanković[1], Peđa Janaćković[1]

[1]*Faculty of Biology, University of Belgrade, Studentski trg 16, 11000, Belgrade, Serbia*

[2]*Department of plant physiology, Institute for Biological Research "Siniša Stanković", National Institute of Republic of Serbia, University of Belgrade, Bulevar despota Stefana 142, 11060 Belgrade, Serbia*

[3]*Faculty of Chemistry, University of Belgrade, Studentski trg 12-16, 11000, Belgrade, Serbia*

#these authors contributed equally to this work

***Corresponding author:**

Dr. Ivica Dimkić, Associate Research Professor

E-mail: ivicad@bio.bg.ac.rs

Tel: +381 (0) 11 2186 635

Fax: +381 (0) 11 2638 500

Table S1 Quantified phenolics and their yield (mg/kg) in *Centaurea calcitrapa* extracts

Compounds	MeOH	EtOH	EtOAc	Me_2CO	DCM:MeOH
Gallic acid	0.09	0.09	0.05	0.06	0.07
Protocatechuic acid	0.75	0.87	0.28	0.90	0.89
Chlorogenic acid	5.98	8.15	0.12	7.32	4.33
p-Hydroxybenzoic acid	2.53	1.78	2.07	3.51	3.52
Gentisic acid	0.42	0.42	0.16	0.25	0.33
Aesculetin	0.06	0.09	0.01	0.18	0.09
Caffeic acid	0.14	0.12	0.05	0.23	0.16
p-Coumaric acid	0.03	0.01	0.01	0.05	0.04
Quercetin-3-O-glucoside	0.08	0.12	ND	0.26	0.18
Ferulic acid	1.33	0.97	0.09	0.54	0.86
Isorhamnetin-3-O-glucoside	0.03	0.10	NF	0.18	0.12
Kaempferol-3-O-glucoside	1.27	1.81	0.02	2.43	1.36
Luteolin	0.10	0.12	0.04	0.69	0.21
Apigenin	1.00	1.00	0.73	2.45	1.30
Kaempferol	0.03	0.03	ND	0.47	0.12
Chrysoeriol	0.62	0.49	0.78	1.55	0.91
Phenolic yield per extract	14.45	16.17	4.41	21.06	14.51

ND – not detected

Table S2 Proposed metabolites and *m/z* peak areas in five different *Centaurea calcitrapa* extracts using UPLC–MS/MS^4 analysis

No	t_R, min	Compound name	MeOH	EtOH	EtOAc	Me_2CO	DCM:MeOH
		Phenolic acids and derivatives					
**1**	2.35	**Gallic acid***	1808503	1613914	524081	1316003	1018021
**2**	3.98	**Dihydroxybenzoyl hexoside**	47197974	48261414	ND	17920103	15603464
**3**	4.32	**Protocatechuic acic***	10550738	12245439	3912666	12727683	12628971
**4**	4.52	**Dihydroxybenzoic acid pentosyl-hexoside**	8702675	13234558	ND	3997265	1198917
**5**	4.60	**Caffeoyl-quinic acid isomer**	20959972	28749302	ND	13490632	9530743
**6**	4.64	**Feruloyl-quinic acid hexoside isomer 1**	1514987	3908647	ND	1372531	323791
**7**	4.71	**Caffeoyl-hexaric acid**	141507	2031761	ND	1002798	ND
**8**	4.83	**Caffeoyl-quinic acid hexoside**	750411	5858176	ND	1815991	88495
**9**	5.15	**Feruloyl-hexaric acid**	2026949	12250436	ND	9013133	94835
**10**	5.17	**Coumaroyl-quinic acid isomer 1**	3292118	2915915	ND	1792204	1973090
**11**	5.24	**Chlorogenic acid***	44766374	61158845	310945	54916883	36449909
**12**	5.26	**Feruloyl-quinic acid hexoside isomer 2**	2182085	2882306	ND	882168	707013
**13**	5.27	***p*-Coumaric acid***	4418991	2924366	127838	968709	3075845
**14**	5.29	**Coumaroyl hexoside**	2641434	977111	186033	774905	2093784
**15**	5.38	***p*-Hydroxybenzoic acid***	2261897	1630566	1697826	2825858	2719709
**16**	5.47	**Feruloyl-quinic acid isomer 1**	52151489	52829900	2413043	38395534	41954435
**17**	5.53	**Gentisic acid***	3396779	3592408	1275552	2061132	2754466
**18**	5.54	**Feruloyl hexoside**	2274398	1988357	119881	1757250	2158363
**19**	5.76	**Aesculetin***	723230	1217763	123443	3103558	1184074
**20**	5.76	**Caffeic acid***	3890751	3217393	1261902	6184634	4763356
**21**	5.89	**Coumaroyl-quinic acid isomer 2**	6349543	9371407	44311	8635329	4301332
**22**	6.18	**Feruloyl-quinic acid isomer 2**	52996767	60084202	3311831	62700948	46182061
**23**	6.30	**Coumaroyl-quinic acid isomer 3**	3425210	4896540	ND	2904467	1792620
**24**	6.49	**Feruloyl-quinic acid isomer 3**	10746649	12209981	93337	7325668	6390873
**25**	6.82	**Ferulic acid***	8895558	5687297	650385	2720269	5890904
**26**	6.82	**Feruloyl pentoside**	5803957	3772826	69433	3156979	2626886
**27**	6.89	**Feruloyl-isocitric acid**	2916784	9678837	ND	5819261	330630
**28**	7.62	**Caffeoyl-feruloyl-quinic acid isomer 1**	754637	1043814	ND	2455260	245268
**29**	7.72	**Dihydroxybenzoyl-feruloyl acid hexoside**	3060868	2834917	ND	2181324	1175350
**30**	7.97	**Caffeoyl-feruloyl-quinic acid isomer 2**	438147	1283691	ND	2777902	153305

Table S2 (continuation)

	Flavonoid glycosides and aglycones						
**31**	5.57	**Apigenin 6,8-di-*C*-hexoside**	591418	1762992	ND	1257254	220881
**32**	6.56	**Apigenin 8-*C*-hexoside**	ND	246675	266332	823126	137873
**33**	6.67	**Quercetin 3-*O*-glucoside***	1158241	1978351	ND	3804026	2722666
**34**	6.70	**Quercetin 3-*O*-hexuronide**	9933463	13359953	ND	16166516	2190876
**35**	6.72	**Scutellarein 7-*O*-hexuronide**	17480588	21322751	ND	22021542	7750394
**36**	6.99	**Apigenin 7-*O*-(6"-rhamnosyl)-hexoside**	862938	4953652	ND	3703384	ND
**37**	7.10	**Kaempferol 3-*O*-glucoside***	31370885	50857595	1687204	63913996	33201527
**38**	7.19	**Isorhamnetin 3-*O*-glucoside***	368427	1482324	ND	2521302	1758075
**39**	7.23	**Apigenin 7-*O*-hexoside**	935310	1352575	ND	3867416	1669282
**40**	7.29	**Apigenin 7-*O*-hexuronide**	43243327	47705052	221591	52330235	28212474
**41**	7.45	**Hispidulin 7-*O*-hexuronide**	7827514	14058864	ND	11303367	4069819
**42**	7.48	**Jaceosidin 7-*O*-hexoside**	2242013	1738629	4875494	1873140	3165430
**43**	7.72	**Kaempferide 3-*O*-hexuronide**	2675815	9099127	ND	6685887	1914505
**44**	7.80	**Kaempferol 3-*O*-acetyl-hexoside**	147372	442841	ND	3114151	123101
**45**	8.10	**Scutellarein**	194945	391814	41522	7006289	157754
**46**	8.68	**Luteolin***	2959321	3615526	1119456	20809728	6387996
**47**	8.81	**Nepetin**	33608625	24399170	57497932	51379879	42297789
**48**	9.54	**Apigenin***	35012456	34883431	26414864	79726361	44508046
**49**	9.70	**Kaempferol***	1910303	1705589	ND	11167828	3553657
**50**	9.72	**Chrysoeriol***	14915798	12133372	20320267	28569714	19602270
**51**	9.74	**Jaceidin**	1102448	1817773	9044127	1149833	3451044
**52**	9.97	**Jaceosidin**	24977383	24763651	39575816	26789180	29505398
**53**	10.03	**Kaempferide**	4213624	3752850	3353072	13265444	6879118
**54**	11.20	**Eupatorin**	38753750	37356743	59963859	34645211	48285312
**55**	11.24	**Centaureidin**	2434347	1863390	4516671	1974934	3242889

*Confirmed using standards, the other compounds were identified according HRMS data and MSn; tR – retention time (min); ND – not detected.
